# Supplementary material for: A versatile, high-efficiency platform for CRISPR-based gene activation
Source: Nat Commun. 2023 Feb 17;14:902. doi: 10.1038/s41467-023-36452-w (PMC9938141; doi:10.1038/s41467-023-36452-w)
Supplement: Supplementary file 1 — Supplementary Information [file 41467_2023_36452_MOESM1_ESM.pdf]

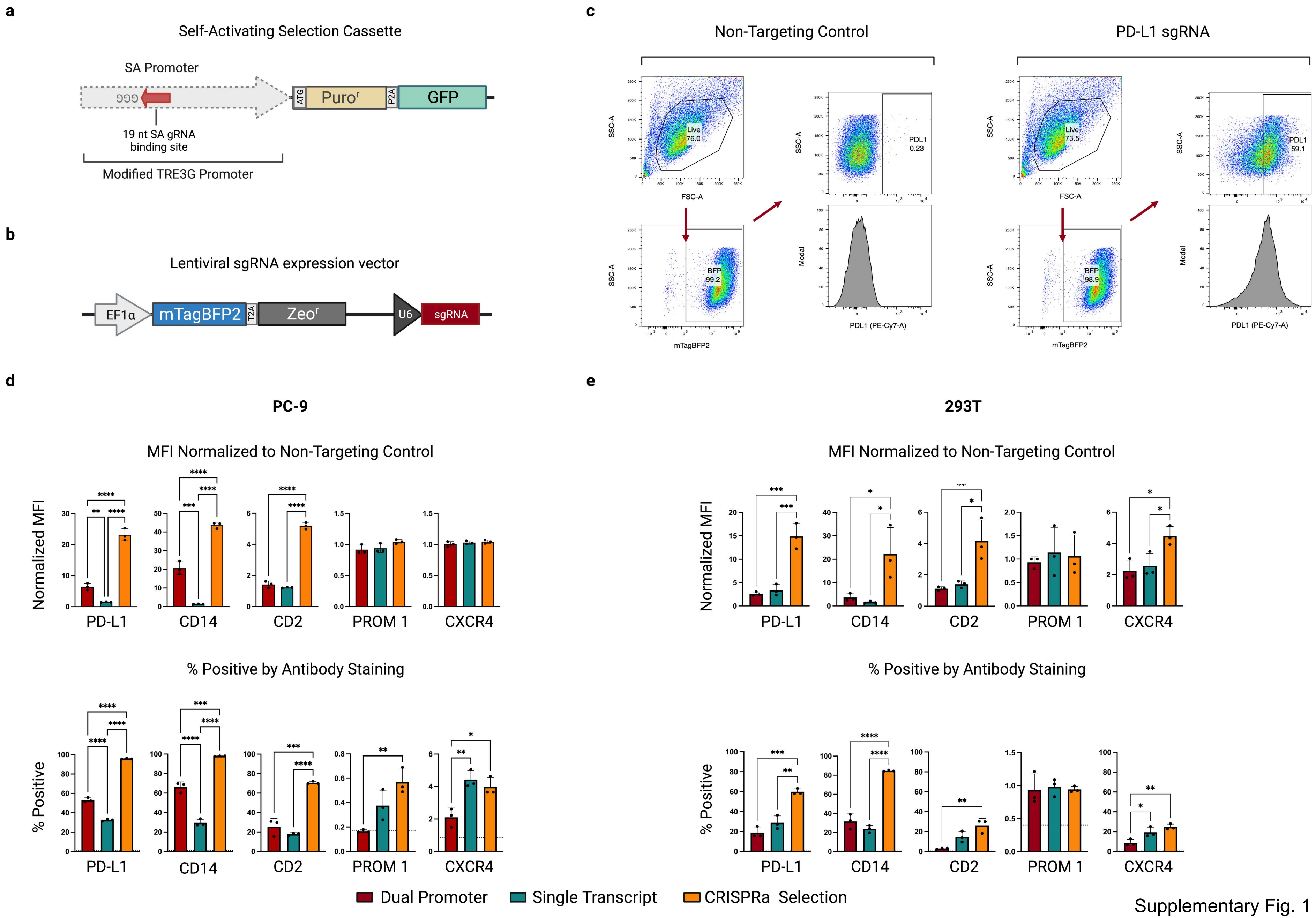

Supplementary Fig. 1

<u>Supplementary Fig. 1:</u> Comparison of CRISPRa piggyBac vector systems in additional cell lines.

a, Schematic representation of the Self Activating (SA) selection cassette. A modified TRE3G promoter with the tetO sites substituted for a single SA gRNA binding site (red arrow) drives expression a Puro^r P2A GFP encoding transcript in cells with functional CRISPR activation machinery and SA gRNA. **b,** Schematic representation of the lentiviral sgRNA expression vector containing a mTagBFP2 marker and Zeocin resistance (Zeo^r) selection cassette. **c,** Representative gating strategy used for all flow cytometric analyses of CRISPRa populations lentivirally transduced with gene targeting sgRNAs or non-targeting controls (left) or *PD-L1* targeting sgRNA (right). Live populations were identified as indicated based on SSC-A (side scatter) and FSC-A (forward scatter) profiles and sgRNA expressing cells were identified by expression of the mTagBFP2 fluorescent protein. Positive population gates were defined in a control sample stained in parallel. **d,** Flow cytometric analysis of CRISPRa mediated gene expression in cell populations generated with three CRISPRa piggyBac systems utilizing distinct selection strategies (Fig.1). Analysis performed 14-25 days post lentiviral transduction of sgRNAs complementary to the promoter proximal regions of the indicated genes (*PD-L1, CD14, CD2, PROM1* or *CXCR4*) for PC-9, or **e,** 293T cells (note: **d**- PROM1 and CXCR4 % and **e**- PROM1 % y-axis scaled to highlight statistically significant differences). Median fluorescence intensity (MFI) was normalized to MFI of an antibody-stained sample expressing a non-targeting gRNA (top). Percent antibody positive is presented (bottom) and background staining from a control sample expressing a non-targeting gRNA is indicated with a dashed horizontal line for each gene. Cell populations generated in triplicate (n=3 biologically independent samples) and sgRNAs infected in duplicate (n=2 technical replicates per biological sample) and averaged. Statistical comparison was performed by an unpaired 1-way ANOVA adjusted for multiple comparisons. Data are presented as mean values +/- SD. * $p<0.05$, ** $p<0.01$, *** $p<0.001$, **** $p< 0.0001$. Source data are provided as a Source Data file.

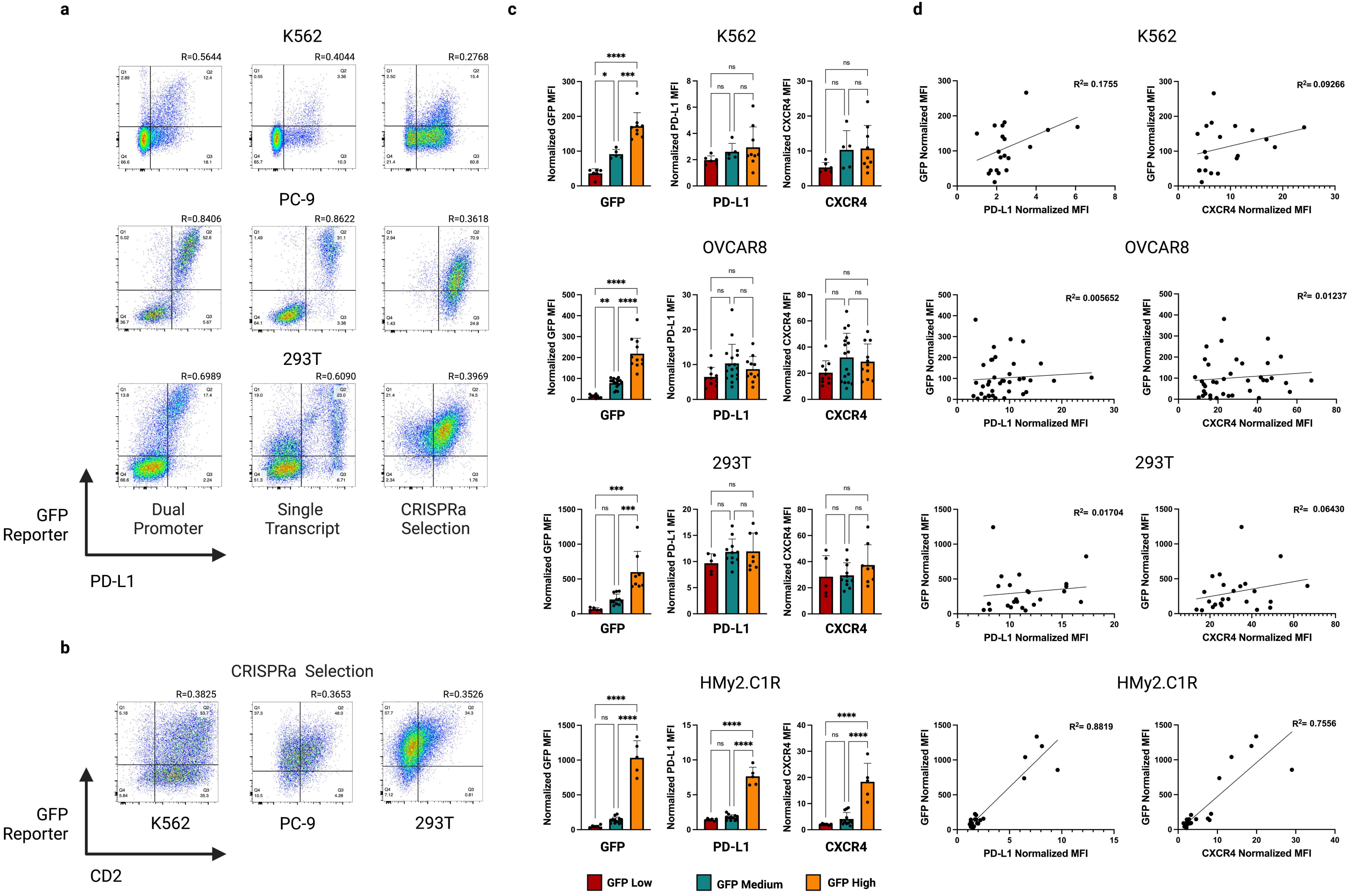

Supplementary Fig. 2

Supplementary Fig. 2: An integrated CRISPRa dependent GFP reporter is an inconsistent marker of CRISPRa efficiency across multiple cell lines.

a, Flow cytometric analysis of GFP CRISPRa reporter vs endogenous *PD-L1* target gene activation across three CRISPRa piggyBac formats in three cell lines (Fig. 1). Pearson correlation values (R) between the fluorescence intensity of GFP and PD-L1 are shown at the top right of each panel. **b**, GFP CRISPRa reporter vs endogenous *CD2* activation in three cell lines engineered with an EF1α-CRISPRa-sel piggyBac. Pearson correlation values between the fluorescence intensity of GFP and CD2 are shown at the top right of each panel. **c**, Flow cytometric analysis of GFP CRISPRa reporter vs two endogenous CRISPRa target genes (*PD-L1, CXCR4*) in clonal lines derived from CRISPRa-sel populations pre-sorted on GFP expression using fluorescence activated cell sorting (FACS) in four distinct cell line backgrounds. Bar graphs of GFP median fluorescence intensity (MFI) in clones normalized to parental cell line (left). Each data point represents the normalized MFI value of an individual clone. Target gene expression in engineered clones infected with an endogenous gene targeting sgRNA (*PD-L1* or *CXCR4*) and normalized to non-targeting control sgRNA (middle/right). Data are presented as mean values +/- SD. **d**, Scatter plots showing correlation of normalized MFI of GFP vs endogenous target gene MFI for each individual clone. R^2 for simple linear regression analysis indicated. Number of clones analyzed per cell line: K562 n=20, OVCAR8 n=38, 293T n=24, and HMy2.C1R n=23. * $p<0.05$, ** $p<0.01$, *** $p<0.001$, **** $p< 0.0001$. Source data are provided as a Source Data file.

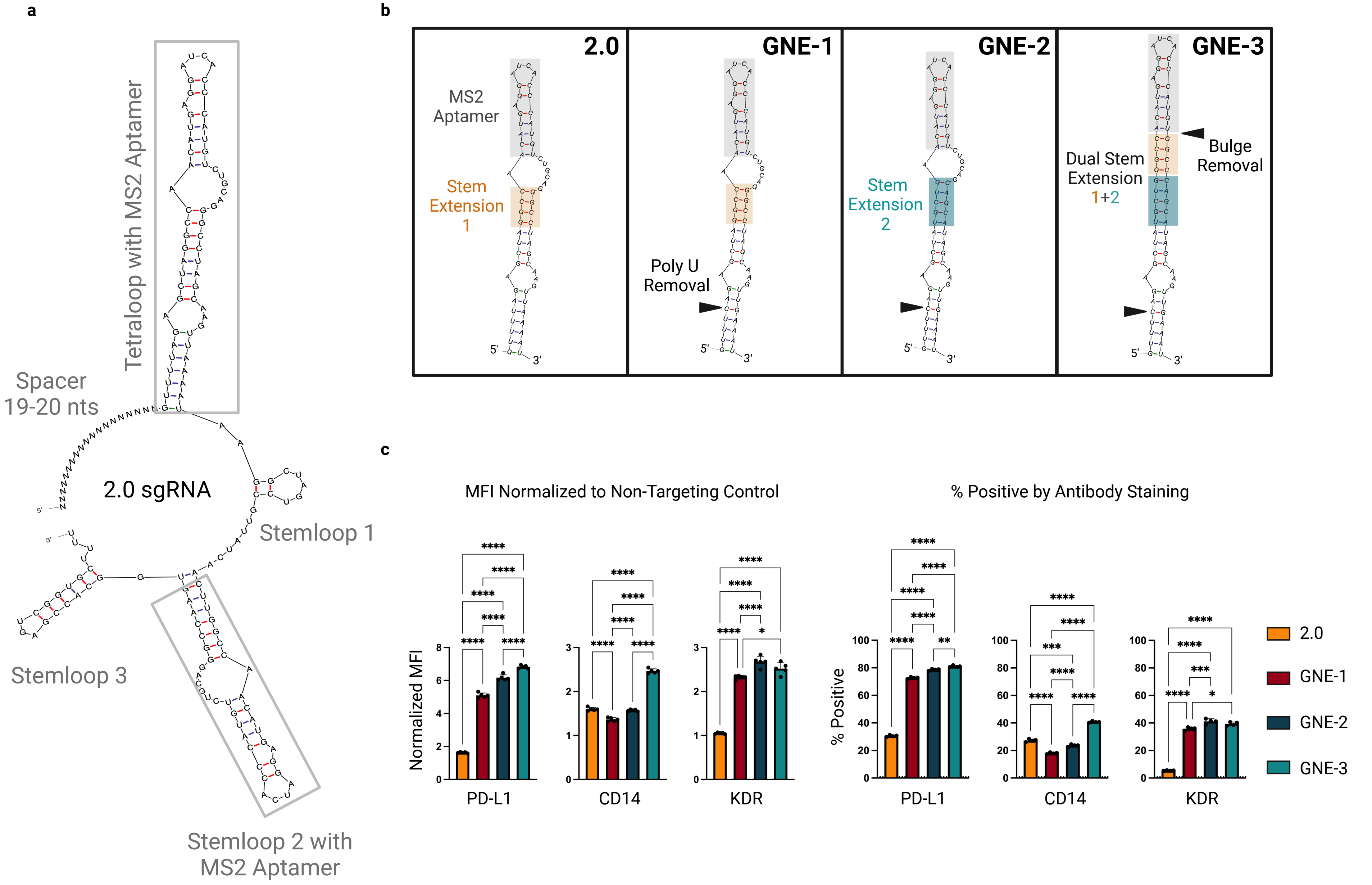

Supplementary Fig. 3

<u>Supplementary Fig. 3:</u> CRISPR activation efficiency of sgRNAs containing scaffold structural modifications.

a, Structure of the 2.0 sgRNA[13] with a modified MS2 aptamer containing tetraloop (black box) and stemloop 2 (gray box). **b,** Enlargement of tetraloop structure with highlighted sequence modifications in the alternate scaffolds evaluated. **c,** Flow cytometry data comparing activation efficiency of the four scaffold formats in a K562 EF1α-CRISPRa-sel population. Cell populations were lentivirally transduced with the sgRNAs targeting 3 endogenous gene targets (*PD-L1, CD14* or *KDR*) and analyzed 7 days post infection. Data represented as median fluorescence intensity (MFI) normalized to a cell population infected with a non-targeting sgRNA (left) or percentage positive (right) with non-targeting sgRNA represented by a dashed horizontal line. n=4 electroporation replicates per condition. Data are presented as mean values +/- SD. Statistical comparison was performed by an unpaired 1-way ANOVA adjusted for multiple comparisons. * p<0.05, ** p<0.01, *** p<0.001, **** p< 0.0001. KDR-Kinase Insert Domain Receptor (VEGF2R/FLK1). Source data are provided as a Source Data file.

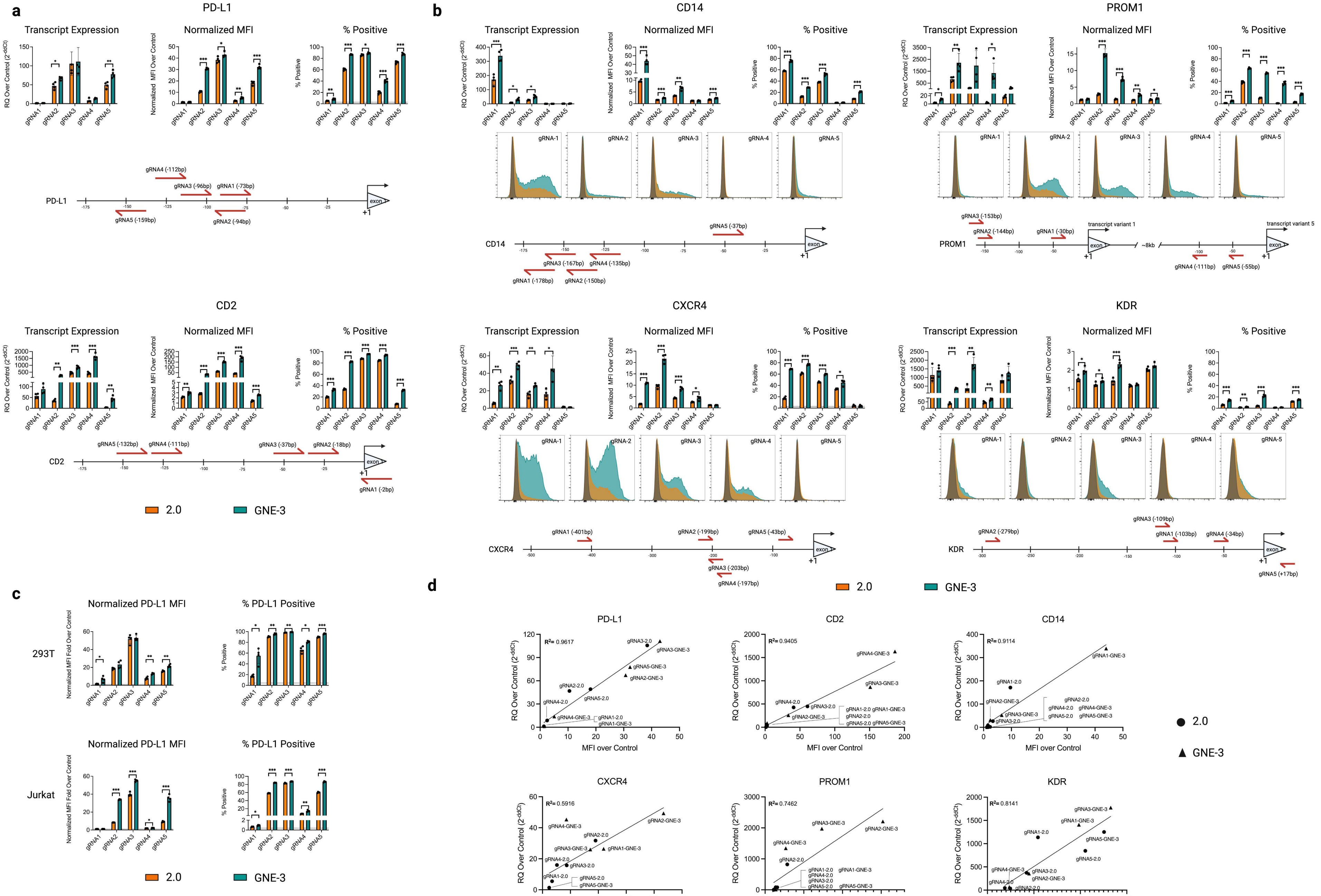

Supplementary Fig. 4

<u>Supplementary Fig. 4:</u> sgRNA activation efficiency of guides in the 2.0 or GNE-3 scaffold context.

a,b, CRISPRa target gene activation by sgRNAs in a 2.0 (orange) or GNE-3 (teal) sequence context in a K562 EF1α-CRISPRa-sel population. **(a)** Expression of *PD-L1* (upper) or *CD2* (lower) target genes assessed by qRT-PCR (left bar plots) and relative to a non-targeting control. Target gene expression assessed by flow cytometry and displayed by median fluorescence intensity (MFI) normalized to a non-targeting control (middle bar plot) or percent target positive (right bar plot). Background percent positive using a non-targeting control sgRNA indicated by a horizontal dashed line. The position of guide RNA binding relative to the transcription start site (TSS) for each gene is indicated below. **(b)** Activation of 4 additional target genes with 2.0 or GNE-3 sgRNAs as assessed by transcript expression (top left), or flow cytometry (Normalized MFI-center; percent positive right or representative histograms, bottom panels) (note: **b**- KDR % y-axis scaled to highlight statistically significant differences). Guide position for each gene relative to the TSS indicated below. **c,** CRISPR-mediated activation of *PD-L1* in two additional cell lines (293T-top and Jurkat-bottom) by sgRNAs in a 2.0 or GNE-3 sequence context. *PD-L1* expression assessed by flow cytometry and represented as MFI normalized to a non-targeting control (left) or percentage PD-L1 positive (right). Percent PD-L1 positive of cells infected with a non-targeting sgRNA represented by a horizontal dashed line. **d,** Scatter plots showing correlation of protein expression (normalized MFI) and transcript expression (qRT-PCR) for each sgRNA evaluated. R squared for simple linear regression analysis indicated. Data are presented as mean values +/- SD. Statistical significance determined by a 2-tailed Student's t-test assuming unequal variance. n=3 biologically independent samples with n=2 technical replicates per biological sample. * $p<0.05$, ** $p<0.01$, *** $p<0.001$. RQ-Relative Quantity. Source data are provided as a Source Data file.

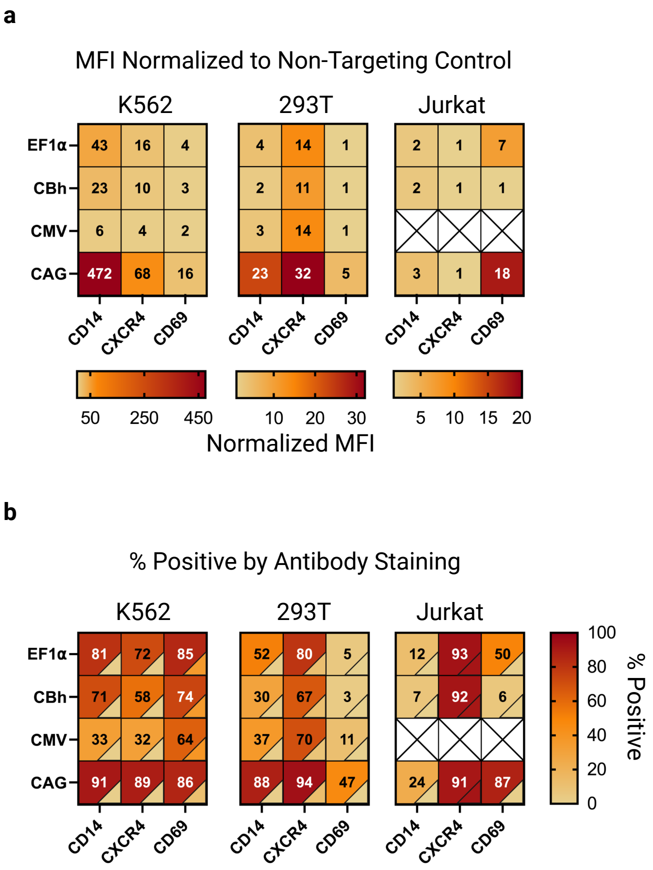

Supplementary Fig. 5

<u>Supplementary Fig. 5:</u> **Extended CRISPRa-sel promoter optimization and application in K562, 293T and Jurkat cell lines.**

a,b Heatmap representing endogenous gene activation of 3 target genes in K562, 293T or Jurkat populations engineered with CRISPRa-sel piggyBac vectors driven by the indicated promoters (EF1α , CBh, CMV or CAG). Cells were infected with lentivirus encoding dual GNE-3 sgRNAs targeting the indicated genes (*CD14, CXCR4* or *CD69*) and assayed by flow cytometry 14 days post-infection/Zeocin selection. (**a**) Median fluorescence intensity (MFI) normalized to a stained cell population infected with a non-targeting control sgRNA in K562 (left), 293T (center) or Jurkat (right). Colorimetric scale for each cell line indicated. (**b)** Percentage of the indicated cell populations positive by antibody staining. Percent positive of stained cell populations expressing a non-targeting sgRNA represented colorimetrically in the lower right corner of each cell. CRISPRa cell populations were generated in triplicate (n=3 biologically independent samples) and infected with indicated sgRNAs in technical duplicates (n=2 technical replicates per biological sample) and averaged. Data are presented as mean values. Source data are provided as a Source Data file.

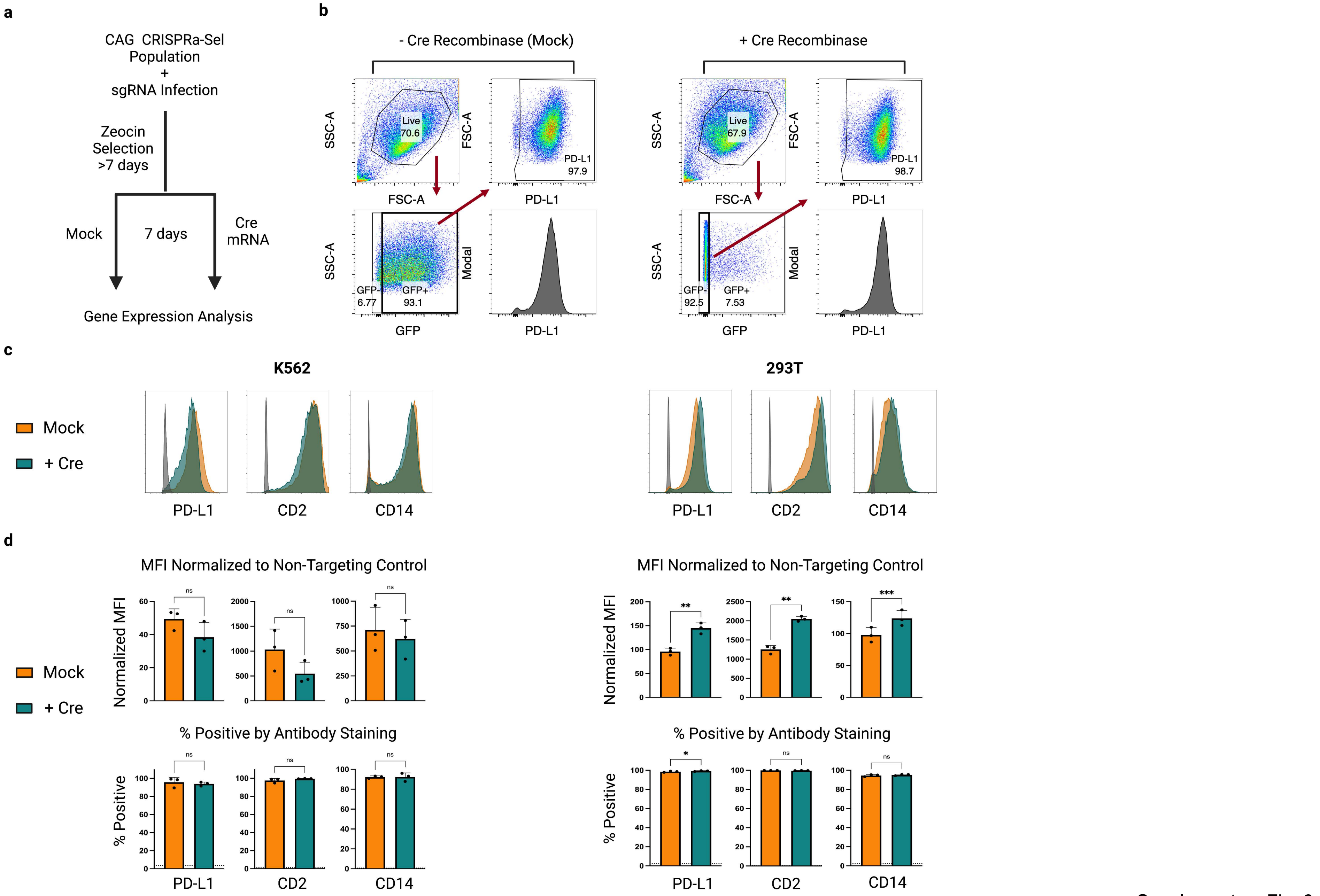

Supplementary Fig. 6

<u>Supplementary Fig. 6:</u> Cre-mediated removal of the CRISPRa-sel cassette shows minimal effect on short term CRISPR-mediated gene activation

a, Experimental overview of Cre-mediated CRISPRa-sel cassette removal. CRISPRa-sel populations were transduced with indicated gene activating sgRNAs. sgRNA expressing cells were enriched under Zeocin selection for a minimum of 7 days. Enriched cell populations were subsequently treated with Cre mRNA to remove the CRISPRa-sel cassette or mock treated. Expression of *PD-L1*, *CD2* and *CD14* evaluated by flow cytometry a minimum of 14 days post infection. **b,** Gating strategy to identify cells containing the CRISPRa-sel cassette (GFP+ left) or cells which have undergone Cre-mediated CRISPRa-sel removal (GFP- right) in a representative 293T cell population. Bold gate indicates the populations analyzed for gene expression in the mock treated (left) or Cre treated (right) populations. **c**, Gene expression for indicated genes (*PD-L1*, *CD2* or *CD14*) displayed by representative overlaid flow cytometry histograms in populations mock treated (orange) or treated with Cre mRNA (teal). Populations transduced with a control sgRNA indicated in gray. K562 (left) and 293T (right) CAG-CRISPRa-sel populations were evaluated. **d**, Median fluorescence intensity (MFI) was normalized to MFI of an antibody-stained sample expressing a non-targeting sgRNA (top). Percent antibody positive is presented (bottom) and background staining from a control sample expressing a non-targeting sgRNA is indicated with a dashed horizontal line for each gene. n=3 biologically independent samples, representative sample shown in **b,c**. Data are presented as mean values +/- SD. Statistical significance determined by a paired 2-tailed t-test. ns $p>0.05$, * $p<0.05$, ** $p<0.01$, *** $p<0.001$. Source data are provided as a Source Data file.

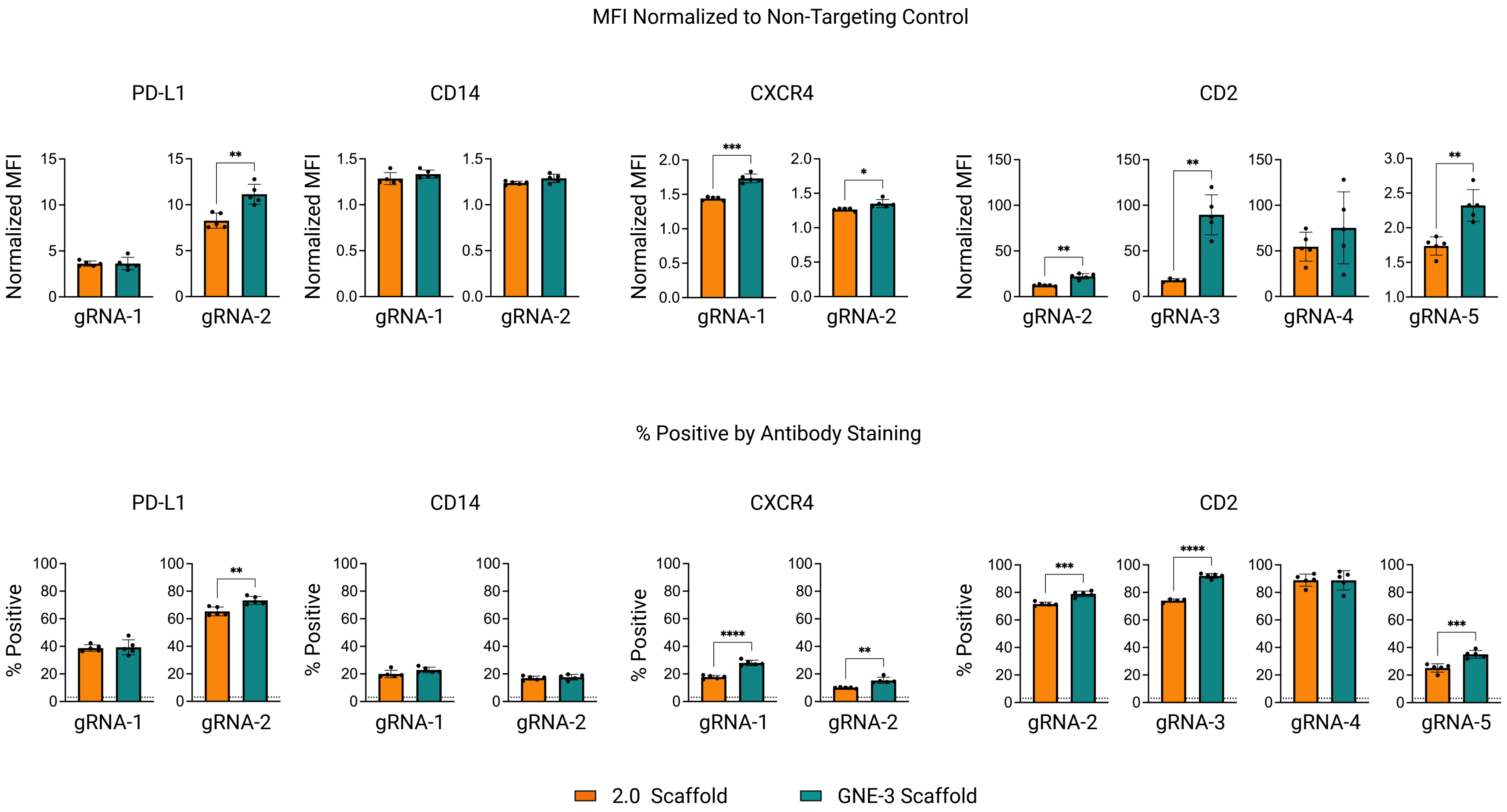

Supplementary Fig. 7

Supplementary Fig. 7: **Activation efficiency of synthetic, modified sgRNAs with a 2.0 or GNE-3 scaffold.**

Evaluation of CRISPR-mediated gene activation in a CAG-CRISPRa-sel engineered K562 population electroporated with modified synthetic sgRNAs in a 2.0 (orange) or GNE-3 (teal) scaffold context. Activation of 4 target genes (*PD-L1*, *CD14*, *CXCR4* or *CD2*) by flow cytometry 3 days post sgRNA electroporation. Data represented as normalized median fluorescence intensity (top) or percent positive observed by antibody staining (bottom). Background antibody staining indicated by a horizontal dashed line. (note: *CD2* gRNA-5 MFI y-axis scaled to highlight statistically significant differences). n=5 electroporation replicates per condition. Data are presented as mean values +/- SD. Statistical significance determined by an unpaired 2-tailed, t-test with a Welch's correction. * $p<0.05$, ** $p<0.01$, *** $p<0.001$, **** $p<0.0001$. Source data are provided as a Source Data file.

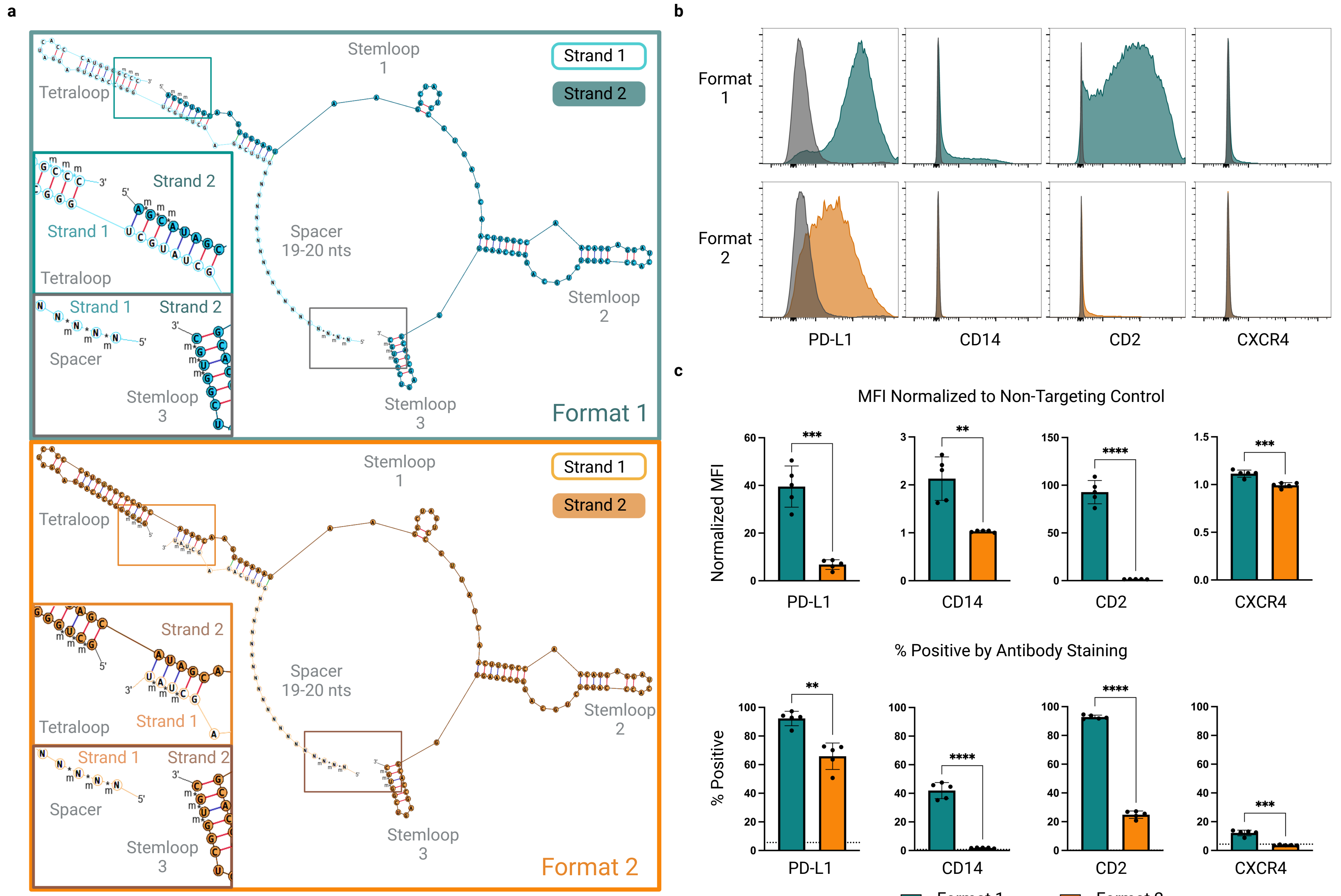

Supplementary Fig. 8

<u>Supplementary Fig. 8:</u> Comparison of alternate synthetic, 2-part GNE-3 guide RNA formats.

a, Diagrams of alternate 2-part, modified, GNE-3 scaffold containing guide RNA structures. Format 1 (teal) encodes a spacer and the majority of the MS2 containing tetraloop on strand 1. Strand 2 of this format encodes the remainder of the tetraloop as well as stemloop 1, stemloop 2 with a second MS2 aptamer and stemloop 3. Strand 1 of the alternate Format 2 structure (orange) encodes a spacer sequence and a short 3' sequence predicted to anneal to the strand 2 scaffold containing the GNE-3 tetraloop and stemloops 1-3. Inset panels indicate the 2'-O-methyl (m) and phosphorothioate (*) linkers on the distal ends of each strand. **b-c**, Evaluation of alternate formats in CAG-CRISPRa-sel engineered K562 populations and 3 days post gRNA electroporation. Expression of indicated endogenous target genes (*PD-L1, CD14, CD2* or *CXCR4*) were evaluated by flow cytometry and presented as **b**, representative histograms overlaid with non-targeting control electroporated populations (gray) or **c,** summarized as normalized median fluorescence intensity (top) or percentage target gene positive (bottom). Background staining indicated by a horizontal dashed line. n=5 technical replicates per condition. Data are presented as mean values +/- SD. Statistical significance determined by an unpaired 2-tailed t-test with a Welch's correction. * $p<0.05$, ** $p<0.01$, *** $p<0.001$, **** $p<0.0001$. Source data are provided as a Source Data file.

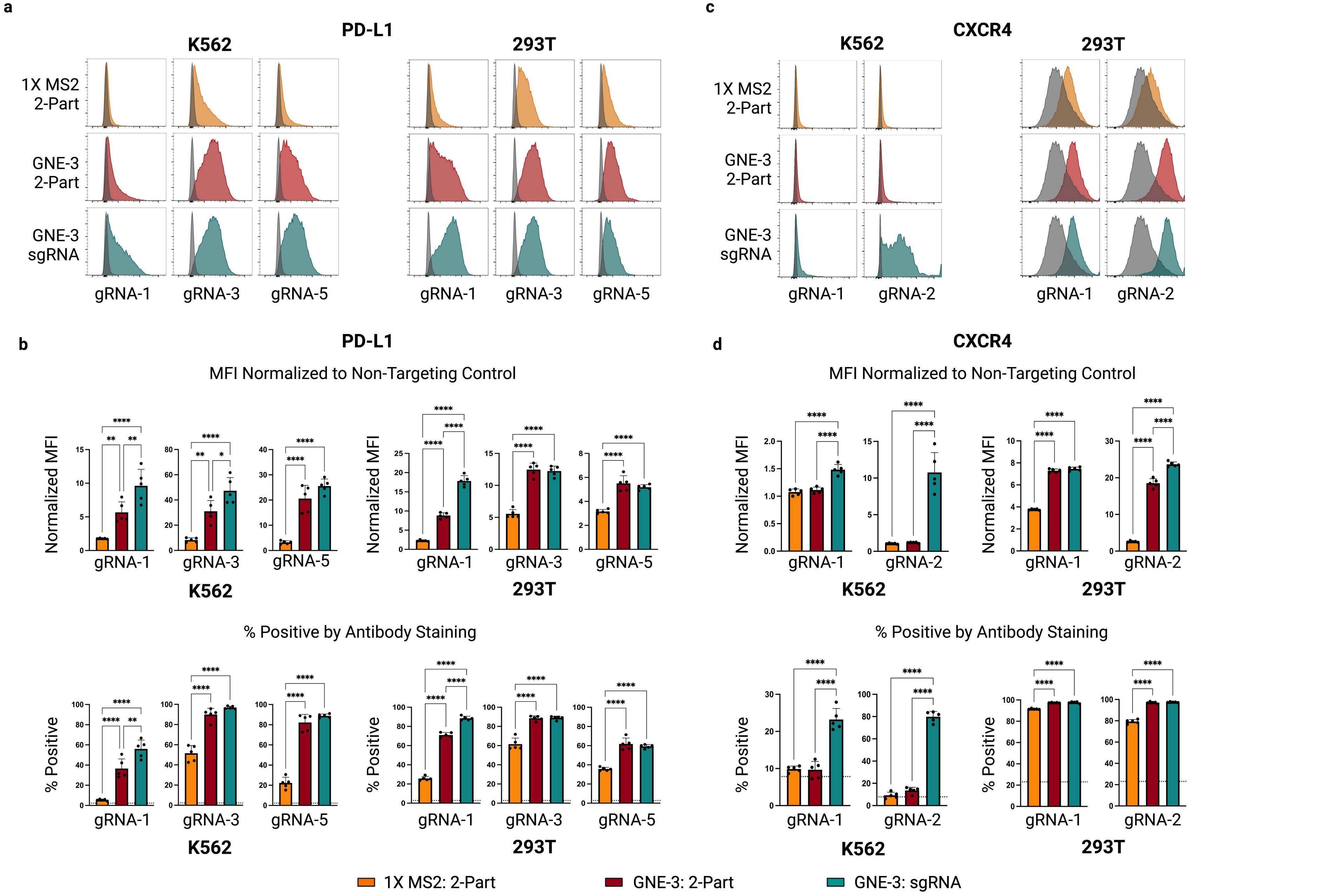

Supplementary Fig. 9

Supplementary Fig. 9: Evaluation of CRISPRa synthetic guide formats in an expanded cell panel.

a-d, Target gene activation comparing three different synthetic guide RNA formats, 1X MS2 2-Part (orange), GNE-3 2-Part (maroon) and GNE-3 sgRNA (teal) as illustrated in Fig. 4a, across CRISPRa-sel K562 and 293T cell populations. **(a)** *PD-L1* and **(c)** *CXCR4* activation assessed by flow cytometry of antibody-stained cell populations 3 days post gRNA electroporation (K562) or transfection (293T). Representative histograms for each gene targeting gRNA are shown, overlaid with histograms for the non-targeting gRNA control (gray). Median fluorescence intensity for each gene targeting gRNA normalized to the non-targeting gRNA control for (**b**, top) *PD-L1* and (**d**, top) *CXCR4*. Percentage of cells positive by antibody staining for PD-L1 (**b**, bottom) or CXCR4 (**d**, bottom), with background staining indicated by a horizontal dashed line. (note: **d**- *CXCR4* gRNA-1 MFI y-axis scaled to highlight statistically significant differences). n=5 electroporation/transfection replicates per condition. Data are presented as mean values +/- SD. Statistical comparison was performed by an unpaired 1-way ANOVA adjusted for multiple comparisons. * p<0.05, ** p<0.01, **** p<0.0001. Source data are provided as a Source Data file.
