# Supplementary material for: A versatile, high-efficiency platform for CRISPR-based gene activation
Source: Nat Commun. 2023 Feb 17;14:902. doi: 10.1038/s41467-023-36452-w (PMC9938141; doi:10.1038/s41467-023-36452-w)
Supplement: Supplementary file 2 — Reporting Summary [file 41467_2023_36452_MOESM2_ESM.pdf]

nature portfolio

Corresponding author(s): Amy Heidersbach, Benjamin Haley

Last updated by author(s): Jul 20, 2022

Reporting Summary

Nature Portfolio wishes to improve the reproducibility of the work that we publish. This form provides structure for consistency and transparency in reporting. For further information on Nature Portfolio policies, see our Editorial Policies and the Editorial Policy Checklist.

Statistics

For all statistical analyses, confirm that the following items are present in the figure legend, table legend, main text, or Methods section.

n/a	Confirmed	
☐	☒	The exact sample size (*n*) for each experimental group/condition, given as a discrete number and unit of measurement
☐	☒	A statement on whether measurements were taken from distinct samples or whether the same sample was measured repeatedly
☐	☒	The statistical test(s) used AND whether they are one- or two-sided<br>*Only common tests should be described solely by name; describe more complex techniques in the Methods section.*
☒	☐	A description of all covariates tested
☐	☒	A description of any assumptions or corrections, such as tests of normality and adjustment for multiple comparisons
☐	☒	A full description of the statistical parameters including central tendency (e.g. means) or other basic estimates (e.g. regression coefficient) AND variation (e.g. standard deviation) or associated estimates of uncertainty (e.g. confidence intervals)
☐	☒	For null hypothesis testing, the test statistic (e.g. *F*, *t*, *r*) with confidence intervals, effect sizes, degrees of freedom and *P* value noted<br>*Give P values as exact values whenever suitable.*
☒	☐	For Bayesian analysis, information on the choice of priors and Markov chain Monte Carlo settings
☒	☐	For hierarchical and complex designs, identification of the appropriate level for tests and full reporting of outcomes
☐	☒	Estimates of effect sizes (e.g. Cohen's *d*, Pearson's *r*), indicating how they were calculated

Our web collection on statistics for biologists contains articles on many of the points above.

Software and code

Policy information about availability of computer code

Data collection	Flow cytometric data collection performed on BD Celesta, BD Fortessa or BD Symphony machines using FACSDIVA v8/v9 acquisition software. ABI QuantStudio 7 Flex real time PCR system.
Data analysis	Relative quantification (2^-ΔΔCT) analysis was performed by QuantSudio 7 software v2. Flow cytometry data was analyzed initially by FlowJo 2 v10.7/v10.8. Data was subsequently analyzed using PRISM and/or excel software. Bar plots/scatter plots and heatmaps were generated using PRISM v9. Statistical tests performed as indicated in figure legends for each experiment. RNA folding performed using mFold or bifold algorithms.

For manuscripts utilizing custom algorithms or software that are central to the research but not yet described in published literature, software must be made available to editors and reviewers. We strongly encourage code deposition in a community repository (e.g. GitHub). See the Nature Portfolio guidelines for submitting code & software for further information.

Data

Policy information about availability of data

All manuscripts must include a data availability statement. This statement should provide the following information, where applicable:

- Accession codes, unique identifiers, or web links for publicly available datasets
- A description of any restrictions on data availability
- For clinical datasets or third party data, please ensure that the statement adheres to our policy

The authors declare that relevant data supporting the findings of this study are available within the article, its supplementary information, and source files.

Human research participants

Policy information about studies involving human research participants and Sex and Gender in Research.

Reporting on sex and gender	N/A
Population characteristics	N/A
Recruitment	N/A
Ethics oversight	N/A

Note that full information on the approval of the study protocol must also be provided in the manuscript.

Field-specific reporting

Please select the one below that is the best fit for your research. If you are not sure, read the appropriate sections before making your selection.

☒ Life sciences ☐ Behavioural & social sciences ☐ Ecological, evolutionary & environmental sciences

For a reference copy of the document with all sections, see nature.com/documents/nr-reporting-summary-flat.pdf

Life sciences study design

All studies must disclose on these points even when the disclosure is negative.

Sample size	Sample size was determined empirically through repeated experimental replicates. Sample size for each experiment indicated in the figure legend, methods or associated text.
Data exclusions	A technical replicate may be missing due to technical problems/sample loss during data collection.
Replication	All comparative studies were performed a minimum of 2 times with representative experiments depicted. Biological and technical replicates for each experiment indicated in figure legend, methods or associated text.
Randomization	No human or animal subjects were included in this study and therefore randomization was not applied.
Blinding	No human or animal subjects were included in this study and therefore blinding was not applied.

Reporting for specific materials, systems and methods

We require information from authors about some types of materials, experimental systems and methods used in many studies. Here, indicate whether each material, system or method listed is relevant to your study. If you are not sure if a list item applies to your research, read the appropriate section before selecting a response.

Materials & experimental systems

n/a	Involved in the study
	☒ Antibodies
	☒ Eukaryotic cell lines
☒	☐ Palaeontology and archaeology
☒	☐ Animals and other organisms
☒	☐ Clinical data
☒	☐ Dual use research of concern

Methods

n/a	Involved in the study
☒	☐ ChIP-seq
	☒ Flow cytometry
☒	☐ MRI-based neuroimaging

Antibodies

Antibodies used

Antibody clone, company and staining protocol listed in supplemental methods 'antibodies'.

Validation

All experiments evaluating CRISPR mediated gene activation utilizing antibody based staining were directly compared to identical cell populations expressing a non-targeting guide RNA control.

Anti-human Prom1/CD133 mouse monoclonal;
Biolegend 372806;
Validated by Biolegend and
Swaminathan SK, et al. 2010. J. Immunol. Methods 361:110.;
https://www.biolegend.com/en-us/products/apc-anti-human-cd133-antibody-13915.

Anti-human CXCR4/CD184 human monoclonal;
Miltenyi 130-120-708;
Extended validation performed by Miltenyi;
https://www.miltenyibiotec.com/US-en/products/cd184-cxcr4-antibody-anti-human-reafinity-rea649.html#apc:100-tests-in-200-ul.

Anti-human CD69 mouse monoclonal;
Biolegend 310910;
Validated by Biolegend and
1. Knapp WB, et al. 1989. Leucocyte Typing IV. Oxford University Press. New York.
2. Sakkas LI, et al. 1998. Clin. and Diag. Lab. Immunol. 5:430.
3. Kim JR, et al. 2005. BMC Immunol. 6:3.
4. Verjans GM, et al. 2007. P. Natl. Acad. Sci. USA 104:3496.
5. Lu H, et al. 2009. Toxicol Sci. 112:363.
6. Thakral D, et al. 2008. J. Immunol. 180:7431.
7. Yoshino N, et al. 2000. Exp. Anim. (Tokyo) 49:97.
8. Radtke AJ, et al. 2020. Proc Natl Acad Sci USA. 117:33455-33465.
9. Radtke AJ, et al. 2022. Nat Protoc. 17:378-401.;
https://www.biolegend.com/en-us/products/apc-anti-human-cd69-antibody-1674?GroupID=BLG10036

Anti-human PD-L1 (CD247) mouse monoclonal;
Biolegend 329718 (PE-cy7), Biolegend 329708 (APC);
Validated by Biolegend and
1. Brown J, et al. 2003. J. Immunol. 170:1257.
2. Radziewicz H, et al. 2007. J. Virol. 81:2545.
3. Nakamoto N, et al. 2009. PLoS Pathog. 5:e1000313.
4. Barsoum IB, et al. 2014. Cancer Res. 74:665.
6. Haile, S et al. 2013. J. Immunol. 191:2829.
7. RL M, et al. 2015. PNAS. 112:6506-6514.
8. Mahoney KM, et al. 2015. Cancer Immunol. Res. 3:1308.;
https://www.biolegend.com/en-us/products/pe-cyanine7-anti-human-cd274-b7-h1-pd-l1-antibody-8277 ;
https://www.biolegend.com/en-us/products/apc-anti-human-cd274-b7-h1-pd-l1-antibody-4376 .

Anti-human CD14 mouse monoclonal;
Biolegend 325608;
Validated by Biolegend and
1. McMichael A, et al. 1987. Leucocyte Typing III. Oxford University Press. New York.
2. Knapp W, et al. Eds. 1989. Leucocyte Typing IV. Oxford University Press. New York.
3. Schlossman S, et al. Eds. 1995. Leucocyte Typing V. Oxford University Press. New York.;
https://www.biolegend.com/en-us/search-results/apc-anti-human-cd14-antibody-3953.

Anti-human CD2 mouse monoclonal:
Biolegend 300208;

Validated by Biolegend and
1. Knapp W, et al. Eds. 1989. Leucocyte Typing IV. Oxford University Press. New York.
2. Aversa G, et al. 1987. Transplant. Proc. 19:277.
3. Zaretsky AG, et al. 2009. J. Exp Med. 206:991.
4. Perona-Wright G, et al. 2010. Nat. Immunol. 11:520.
5. Thummler K, et al. 2010. J. Leukoc. Biol. 88:1041.
6. Kap Y, et al. 2009. J. Histochem. Cytochem. 57:1159.
7. Yoshino N, et al. 2000. Exp. Anim. (Tokyo) 49:97.;
https://www.biolegend.com/en-us/products/pe-anti-human-cd2-antibody-820

Anti-human KDR/VEGF2R-2/CD309 mouse monoclonal:
BD 560494 (PE); BD 560495 (Alexa Flour 647);
Validated by BD and
1. Farace F, Massard C, Borghi E, Bidart JM, Soria JC. Vascular disrupting therapy-induced mobilization of circulating endothelial progenitor cells. Ann Oncol. 2007; 18(8):1421-1422.
2. Ferrara N, Houck K, Jakeman L, Leung DW. Molecular and biological properties of the vascular endothelial growth factor family of proteins. Endocr Res. 1992; 13(1):18-32.
3. Yang L, Soonpaa MH, Adler ED, et al. Human cardiovascular progenitor cells develop from a KDR+ embryonic-stem-cell-derived population. Nature. 2008; 453(7194):524-528.
4. Ziegler BL, Valtieri M, Porada GA, et al. KDR receptor: a key marker defining hematopoietic stem cells. Science. 1999; 285:1553-1558.
https://www.bdbiosciences.com/en-us/products/reagents/flow-cytometry-reagents/research-reagents/single-color-antibodies-ruo/pe-mouse-anti-human-cd309-vegfr-2.560494. ; https://www.bdbiosciences.com/en-us/products/reagents/flow-cytometry-reagents/research-reagents/single-color-antibodies-ruo/alexa-fluor-647-mouse-anti-human-cd309-vegfr-2.560495.

Anti-human CXCR4/CD184 mouse monoclonal;
ThermoFisher/eBioscience 17-9999-42;
Validated by ThermoFisher/eBioscience and
Strizki JM, Turner JD, Collman RG, Hoxie J, González-Scarano F. A monoclonal antibody (12G5) directed against CXCR-4 inhibits infection with the dual-tropic human immunodeficiency virus type 1 isolate HIV-1(89.6) but not the T-tropic isolate HIV-1(HxB). J Virol. 1997 Jul;71(7):5678-83.;
https://www.thermofisher.com/antibody/product/CD184-CXCR4-Antibody-clone-12G5-Monoclonal/17-9999-42.

Eukaryotic cell lines

Policy information about cell lines and Sex and Gender in Research

Cell line source(s)

All parental cell lines were sourced from the Genentech cell bank (gCell):
K562
PC-9
293T (GNE293T)
Jurkat
Ovcar8
HMy2.C1R
A375
A549
DLD1
H358
HCT 116
HT-29
MCF-7
MDA-MB-231
RKO

Authentication

Cell lines authenticated by STR profiling as described doi.org/10.1038/nature14397.

Mycoplasma contamination

Cells tested negative for mycoplasma contamination were maintained under mycoplasma-free conditions.

Commonly misidentified lines
(See ICLAC register)

No commonly misidentified lines were used in these studies.

Flow Cytometry

Plots

Confirm that:

- [ ] The axis labels state the marker and fluorochrome used (e.g. CD4-FITC).
- [ ] The axis scales are clearly visible. Include numbers along axes only for bottom left plot of group (a 'group' is an analysis of identical markers).
- [ ] All plots are contour plots with outliers or pseudocolor plots.
- [x] A numerical value for number of cells or percentage (with statistics) is provided.

Methodology

Sample preparation	All antibody/ cell line specific staining methods are provided in supplemental methods 'antibody' tab.<br><br>Prom1 Biolegend clone7 372806 (APC) Pre-wash cells 1 X with staining buffer. Stain: 5ul antibody in 100ul staining buffer. Incubation 20-30min @ 4deg. Wash: 2 X with staining buffer.<br><br>CXCR4 Miltenyi rea649 130-120-708 (APC) Pre-wash cells 1 X with staining buffer. Stain: 2ul antibody in 100ul staining buffer. Incubation 20-30min @ 4deg. Wash: 2 X with staining buffer.<br><br>CD69 Biolegend fn50 310910 (APC) Pre-wash cells 1 X with staining buffer. Stain: 2ul antibody in 100ul staining buffer. Incubation 20-30min @ 4deg. Wash: 2 X with staining buffer.<br><br>PD-L1 Biolegend 29e.2a3 329718 (PE-cy7); 329708 (APC) Pre-wash cells 1 X with staining buffer. Stain: 2ul antibody in 100ul staining buffer. Incubation 20-30min @ 4deg. Wash: 2 X with staining buffer.<br>CD14 Biolegend hcd14 325608 (APC) Pre-wash cells 1 X with staining buffer. Stain: 2ul antibody in 100ul staining buffer. Incubation 20-30min @ 4deg. Wash: 2 X with staining buffer.<br><br>CD2 Biolegend rpa-2.10 300208 (PE) Pre-wash cells 1 X with staining buffer. Stain: 2ul antibody in 100ul staining buffer. Incubation 20-30min @ 4deg. Wash: 2 X with staining buffer.<br><br>KDR BD 89106 560494 (PE), 560495 (Alexa Flour 647) Pre-wash cells 1 X with staining buffer. Stain: 20ul antibody in 100ul staining buffer. Incubation 30-40min @ 4deg. Wash: 2 X with staining buffer.<br><br>CXCR4 ThermoFisher/eBioscience 12G5 17-9999-42 (APC) Pre-wash cells 1 X with staining buffer. Stain: 0.2ul antibody in 50ul staining buffer. Incubation 20-30min @ 4deg. Wash: 2 X with staining buffer.
Instrument	BD Celesta, BD Fortessa or BD Symphony
Software	FACSDIVA v8/v9 was utilized as acquisition software and FlowJo 2 v10.7/v10.8 was used for subsequent analysis
Cell population abundance	Populations were defined by gates established (as indicated Supplementary Fig. 1b) with 2 parameter pseudocolor plots defined in identical control cell lines expressing a non-targeting control guide RNA and stained/collected in parallel. Percent positive and normalized MFI quantified and analyzed as indicated in methods/figure legend. Representative antibody stained populations are displayed by histogram overlaid with the profile of a negative (non-targeting guide) control population stained in parallel for illustrative purposes.
Gating strategy	Gating strategy indicated in Supplementary Fig. 1b. Live cell populations were gated using fsc and ssc profiles. Where relevant, lentivirally transduced cells specifically were examined by gating on mTagBFP2 positive populations. If cell populations were selected to greater than >95% mTagBFP2 positive then this gating step was omitted for some analyses. 'Positive' populations were defined by gates established as indicated with 2 parameter pseudocolor plots (Supplementary Fig. 1b) with identical control cell lines expressing a non-targeting control guide RNA and stained/collected in parallel.

- [x] Tick this box to confirm that a figure exemplifying the gating strategy is provided in the Supplementary Information.
