# Supplementary material for: A versatile, high-efficiency platform for CRISPR-based gene activation
Source: Nat Commun. 2023 Feb 17;14:902. doi: 10.1038/s41467-023-36452-w (PMC9938141; doi:10.1038/s41467-023-36452-w)
Supplement: Supplementary file 3 — Description of Additional Supplementary Files [file 41467_2023_36452_MOESM3_ESM.pdf]

Title: Supplementary Data 1

Description: Reagents Sequences.xlsx
